# Baroreceptors in the Aortic Arch and Their Potential Role in Aortic Dissection and Aneurysms

**DOI:** 10.3390/jcm11051161

**Published:** 2022-02-22

**Authors:** Benedikt Reutersberg, Jaroslav Pelisek, Ahmed Ouda, Olivier de Rougemont, Fabian Rössler, Alexander Zimmermann

**Affiliations:** 1 Department of Vascular Surgery, University Hospital Zurich, 8091 Zurich, Switzerland; jaroslav.pelisek@usz.ch (J.P.); alexander.zimmermann@usz.ch (A.Z.); 2 Department of Cardiac Surgery, University Hospital Zurich, 8091 Zurich, Switzerland; ahmed.ouda@usz.ch; 3 Department of Surgery and Transplantation, University Hospital Zurich, 8091 Zurich, Switzerland; olivier.derougemont@usz.ch (O.d.R.); fabian.roessler@usz.ch (F.R.)

**Keywords:** baroreceptors, ion channels, mechanotransduction, aortic dissection

## Abstract

The arterial baroreflex is a key autonomic regulator of blood pressure whose dysfunction has been related to several cardiovascular diseases. Changes in blood pressure are sensed by specific mechanosensory proteins, called baroreceptors, particularly located in the outer layer of the carotid sinus and the inner curvature of the aortic arch. The signal is propagated along the afferent nerves to the central nervous system and serves as negative feedback of the heart rate. Despite extensive research, the precise molecular nature of baroreceptors remains elusive. Current knowledge assumes that baroreceptors are ion channels at the nerve endings within the outer layer of the arteries. However, the evidence is based mainly on animal experiments, and the specific types of mechanosensitive receptors responsible for the signal transduction are still unknown. Only a few studies have investigated mechanosensory transmission in the aortic arch. In addition, although aortic dissection, and particularly type A involving the aortic arch, is one of the most life-threatening cardiovascular disorders, there is no knowledge about the impact of aortic dissection on baroreceptor function. In this review, we aim not to highlight the regulation of the heart rate but what mechanical stimuli and what possible ion channels transfer the corresponding signal within the aortic arch, summarizing and updating the current knowledge about baroreceptors, specifically in the aortic arch, and the impact of aortic pathologies on their function.

## 1. Introduction

**Figure 1:**
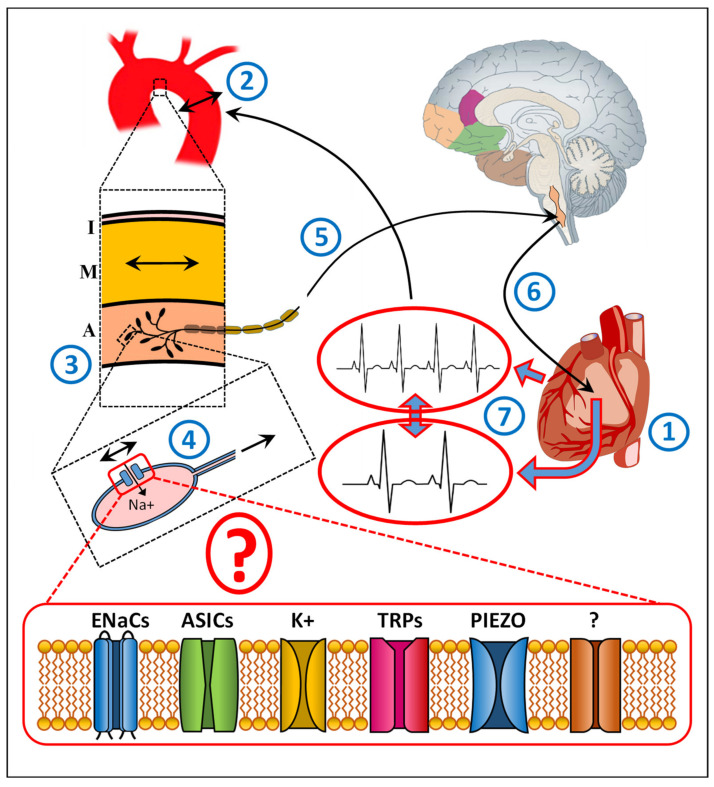
A schematic diagram illustrating the baroreceptor reflex of the aortic arch and the involvement of ion channels. The heart (1) regulates its rate according to the body’s needs. Changes in heart rate (1) alternate blood pressure. The corresponding changes exercise mechanical forces on the aortic wall (2). The changes in shear stress are sensed by specific mechanoreceptors, called baroreceptors, at the endings of sensory nerves innervating the outer wall (adventitia) of aortic arch (3). These mechanical deformations are affecting the mechanosensitive baroreceptors (4), assumed to be specific ion channels, which in turn provide an action potential in the corresponding nerve terminal, which is propagated along the afferent nerve fibers (5) toward the nodose ganglia in the brainstem. The incoming signal is part of the negative feedback system (6) that contributes to the regulation of heart rate (7) to restore heart rate to its normal level. I, Intima, the inner aortic wall layer containing endothelial cells; M, Media, the middle aortic layer containing particularly smooth muscle cells, regulating the vascular tone; A, Adventitia, the outer aortic layer containing the mechanosensory baroreceptors. ENaCs, Epithelial sodium channels, containing four subunits (α, β, γ, and δ) [7,8,9,10,11,12,13,14,15,16]; ASICs, Acid-sensing ion channels [12,13,14,15,16,17,18,19,20,21,22,23,24] belonging to the DEG/ENaC superfamily with at least seven ASIC isoforms; K+, Stretch-sensitive K+ channels, both calcium (Ca)-insensitive and Ca-sensitive [25,26]; TRP, transient receptor potential ion channels [6,27,28,29,30,31]: TRPA (ankyrin 1), TRPC (canonical 1–7), TRPM (melastatin 1–8), TRPN (NOMPC-like 1), TRPP (polycystin 2, 3, 5), TRPML (mucolipin 1–3), and TRPV (vanilloid 1–6): TRPV1, TRPC1, and TRPC3-7; PIEZO1, 2, mechanosensitive ion channels [32,33,34].

In humans, blood pressure is regulated within a tight physiological range. In order to achieve appropriate and fast changes in blood circulation, the cardiovascular system is under the control of the autonomic nervous system. One of the most important mechanisms to sustain circulatory equilibrium is arterial baroreflex [1,2,3]. The changes in blood pressure exercise mechanical force on the arterial wall, which is reflected by shear stress sensed by specific mechanoreceptors, called baroreceptors, at the endings of sensory nerves, which innervate the carotid sinus and aortic arch as well as the pulmonary and coronary arteries and the atria and ventricles [4,5]. Mechanical deformations of the arterial wall are affecting the baroreceptors, which in turn provide an action potential activating the corresponding nerve terminal, which is propagated further on along the afferent nerve fibers toward the brainstem (Figure 1). The incoming signal is part of a negative feedback system of the heart rate [5,6]. The heart rate is regulated by the cardiac sinoatrial node (SAN), which is innervated by sympathetic and parasympathetic autonomic nervous system and changes the R-R interval. By influencing the cells within SAN, nerve impulses and hormones can affect the speed at which the SAN generates an electrical impulse, regulating the heart rate. The parasympathetic input acts via the vagus nerve synapses releasing acetylcholine, which decreases the slope of the pacemaker potential and reduces the heart rate. In the contrary, the sympathetic input acts via postganglionic fibers innervating SAN releasing noradrenaline, increasing the slope of the pacemaker potential and the heart rate. In addition, the heart rate can be modulated indirectly by the renin-angiotensin system. It is to mention that at physiological level the baroreflex is used to quantify its effect on the heart rate. Baroreceptor sensitivity requires beat-to-beat information from blood pressure and the R-R interval. The systolic blood pressure is derived from the systemic arterial pressure but the R-R interval from the electrocardiogram. Furthermore, baroreflex-mediated changes affect heart rate, contractility, and peripheral vascular resistance. However, for simplicity, and because we focus in our review on the molecular nature of baroreceptors, we describe the baroreflex only in context of heart-rate regulation.

Baroreceptors are assumed a type of “pressure sensors” on specific sensory neurons that can detect mechanical stretches within the arterial wall. Thus far, they have been identified particularly at the bifurcation of the external and internal carotid artery and along the inner aortic arch in humans [35]. Electron microscopy revealed the presence of sensory nerves containing the mechanosensory baroreceptors within the tunica adventitia predominantly in an elastic zone [36,37,38,39]. The collagen filaments meander around the nerve terminals and terminate on the surface of the elastic fibers or on the basement membrane of the neuronal endings. Such arrangement of the extracellular matrix ensures large distensibility of this part of the arterial wall containing baroreceptors and provides high sensitivity to changes in intraluminal pressure, thereby facilitating the transmission of the stimulus intensity to sensory nerve terminals. Overall, baroreceptors are localized in regions of higher flexibility, which are composed of more elastin and less collagen. This results in a higher sensitivity [24,38,39,40,41,42]. The specific function of baroreceptors and their high sensitivity are accomplished by a dense network between elastic and collagen fibers terminating at the basement membrane of the sensory neurons. At rest, collagen fibers are preserved in a contractile state [38,39,40,41,42,43]. When the arterial wall is stretched, the network induces collagen unfolding and elastin expansion, leading to mechanical deformation of the baroreceptor terminals via traction at the basement membrane [38,40,42].

Many studies have tried to explore the molecular nature of mechanosensory baroreceptors and to elucidate their role in various cardiovascular diseases [4,5,6,24,35,40,41,42]. However, the precise molecular identity of baroreceptors remains still elusive, as findings of these studies often have been controversial and are based mainly on animal experiments (monkey, mouse, rat, dog, cat, goat, giraffe, pig) [4,6,18,24,32,33,34,35,36,37,38,39,40,41,42,43,44,45,46,47]. Furthermore, much less is known about the role of baroreceptors in the aortic arch. Interestingly, the importance of aortic versus carotid baroreceptors was only mentioned by Timmers et al. [35], stating that aortic baroreceptors might be the dominant ones in the regulation of heart rate [35,48,49]. Thus, it is of uttermost importance, particularly in clinical settings, to elucidate the contribution of baroreceptors in the aortic arch in their regulation of the heart rate in order to apply appropriate drug therapy. Ion channels have already been widely recognized as important therapeutic targets. Progress has been made particularly in specific antibodies targeting selected ion channels [50]. Some of them are already in clinical trials [51]. Such antibodies are not specific against ion channels located in baroreceptors within aortic arch. However, a local delivery, e.g., using injection catheter, would considerably reduce potential side effects and might considerably improve the baroreceptor sensitivity.

It is to note that the aim of our current review was not to highlight the regulation of the heart rate but what mechanical stimuli and what possible ion channels transfer the corresponding signal within the aortic arch.

## 2. Baroreceptor Research in Animals

Recent animal studies suggest that ion channels are most likely involved in the mechanoelectrical transduction performed by arterial baroreceptors. In this context, many ion channels have been investigated, including epithelial sodium (Na+) ion channels (ENaCs), acid-sensing ion channels (ASICs), potassium (K+) ion channels, transient receptor potential (TRP) ion channels, and piezo ion channels. However, whether these channels act directly as mechanosensors or play a role downstream of the mechanotransduction has not yet been elucidated. 

ENaCs are members of the amiloride-sensitive degenerin channel (DEG) family [7,8,9,10,11,41,43]. Four subunits (α, β, γ, and δ) have been detected thus far and are expressed in different tissues [11,12,13,14,15,16]. ENaCs play a significant role in the regulation of water homeostasis and as an Na+ transporter [17]. Recently, they have also been suggested to act as mechanosensors [11,12]. However, their role and function have been mainly investigated in the carotid sinus [8,12,16,17,40,43]. Regarding the few publications focusing on expression of DEG/ENaC in the aortic arch, animal experiments have detected only the β- and γ-subunits [11,24]. DEG/ENaC proteins are found particularly in mechanosensory neurons and in baroreceptor sensory-nerve terminals. The data assume that these proteins might be part of a mechanosensitive ion-channel complex. However, definite evidence that ENaCs serve directly as mechanosensitive baroreceptors is still lacking. Furthermore, despite the recent knowledge, no data regarding the role of ENaCs in the human aortic arch are available thus far. 

ASICs also belong to the DEG/ENaC superfamily [16,17,18,41]. Thus far, seven ASIC isoforms have been found in mammalian nervous systems (1a, 1b, 2a, 2b, 3, 4, and 5) [40,41]. Mutations in ASIC−1, −2, or −3 abolished neurosensory mechanotransduction in deficient peripheral tissue in mice [18,19,20,21,22,23], and both ASIC mRNA and proteins have been detected in murine arterial baroreceptors [24]. Moreover, ASIC−1, −2, and −3 proteins have been detected in nerve fibers and aortic baroreceptor terminals in various animal models [12,46]. No published data, however, exist regarding ASIC in human baroreceptors. 

Stretch-sensitive K+ channels are ion channels selective for potassium cations (K+) and lead to K+ influx following mechanical stress [25,26,41,45]. Two groups of K+ channels have been identified: calcium (Ca)-insensitive and Ca-sensitive. Ca-insensitive K+ channels are activated by osmotic swelling, while Ca-sensitive K+ channels are dependent on steep voltage and Ca concentration. Thus, these ion channels might also serve as mechanosensitive baroreceptors. They have, however, a broad spectrum of action, and again their role in the mechanotransduction of human baroreceptors has not yet been explored.

TRP ion channels belong to a large family of cellular sensors that are able to respond to a plethora of stimuli, including temperature, light, membrane stretching, taste, pain, pheromones, and osmotic stress [24,27,28,29,30,46]. Seven subfamilies have been reported: TRPA (ankyrin 1), TRPC (canonical 1–7), TRPM (melastatin 1–8), TRPN (NOMPC-like 1), TRPP (polycystin 2, 3, 5), TRPML (mucolipin 1–3), and TRPV (vanilloid 1–6) [24,30]. TRPV1, TRPC1, and TRPC3-7 have been detected in animal baroreceptor neurons. Specifically, TRPV1, TRPC1, −3, −4, and −5 are expressed mainly in myelinated and unmyelinated aortic axons and baroreceptor terminals [4,6,31]. TRPC5 channels have been described to be activated in arterial baroreceptors by mechanical stress [6]. All these results originate again from animal experiments. No results describing the potential role of TRP ion channels in human baroreceptors, especially in the aortic arch, have been published so far.

Recently, two novel mechanosensitive ion channels, PIEZO1 and PIEZO2, were described using mouse neuronal N2A cells and Affymetrix microarrays [32]. Mechanical stimuli have directly induced overexpression of these two mechanosensitive ion channels. They seem to have an important biological function of mechanosensation [32,33,34,41,47]. Piezo ion channels are activated by shear stress and play an essential role in airway stretch sensation [32,33,34]. Interestingly, their distribution and expression patterns are quite different. Whereas PIEZO1 is expressed, e.g., in skin, bladder, colon, kidney, and lung, PIEZO2 is highly expressed in particular in sensory dorsal root ganglia. Furthermore, PIEZO1 has been detected in the cellular membrane, thus being a promising candidate for a mechanosensitive baroreceptor [32,41]. The knockout of both PIEZO1 and PIEZO2 in a mouse model completely eliminated the baroreceptor reflex [32]. However, it is not known why both baroreceptors are necessary for proper functionality. Furthermore, they have not been identified in the arterial wall. Thus, their potential role in the baroreceptors within the aortic arch is yet to be elucidated. Again, as with all the other potential mechanosensitive ion channel candidates, these findings are based on mouse experiments and have not yet been confirmed in humans. 

Taken together, a numerous ion channels have been assumed to contribute to arterial baroreflex. Even if some of the above described candidates have been shown in animal experiments to be activated by mechanical stimuli (Figure 1), no data exist confirming whether these ion channels play also a similar role in the aortic arch as mechanosensory baroreceptors, particularly in humans. Furthermore, whether these ion channels are acting directly as stretch sensors or are playing a role further downstream of the mechanotransduction has not yet been elucidated. Even if the baroreceptors seem to have been preserved in evolution among the species, there is still no proof that the same mechanosensitive ion channels supposed to act as baroreceptors described across different animals are expressed and have the same function in humans as well.

## 3. Baroreceptor Research in Humans

Thus far, no histological studies on human baroreceptors have been performed. Only two studies have mentioned anatomical innervation of the aortic arch, dated 80 years ago [52,53]. Studies on baroreceptors in humans have been based so far on physiological approaches, with few of them related to the aortic arch [54,55,56,57,58,59,60,61,62,63]. However, such physiological studies determine baroreflex sensitivity only indirectly by recording electrocardiography and beat-to-beat blood pressure over time, calculating changes in heart rate in response to changes in arterial blood pressure. Consequently, they deliver only scant information about the baroreceptor nature or function for diagnostic or therapeutic purposes. These studies have shown that the calculated baroreceptor sensitivity is markedly affected by age [58,59] and is significantly diminished by patients with arterial hypertension [59], often due to stiffening of the larger elastic arteries, especially the aorta. Additional possible causes include vessel wall remodeling and inflammation. Interestingly, despite its clinical relevance, only one study has attempted to relate the role of baroreceptors with aortic aneurysm pathogenesis [58]. 

In summary, current data suggest that baroreceptors are most likely mechanosensitive ion channels, such as N+ or K+ channels or TRP- or PIEZO-derived channels. Which of them, either a single one or many different working in line, has not yet been elucidated (Figure 1). Furthermore, whether these ion channels are directly responsive to mechanical stimulation or act downstream of the mechanotransduction is still not clear. In addition, the results regarding their molecular nature and function are not yet conclusive and based mainly on animal experiments. Finally, with the exception of the physiological approach, no comparable studies have been performed in humans, particularly in the aortic arch or aneurysm, despite the potential role of baroreceptors in aortic diseases. 

## 4. Baroreceptors and Aortic Dissection

Aortic dissection (AD), in particular acute aortic dissection (AAD), is one of the most lethal cardiovascular diseases even if properly treated [64,65,66,67,68]. Irrespective of the latest novel surgical techniques, mortality remains extremely high (<30%) and is frequently associated with further complications [64,65]. In particular, type A, located in the aortic arch containing mechanosensitive baroreceptors, has high mortality rates of up to 26% in patients who undergo surgery and up to 58% in those treated non-invasively, respectively [67,68,69]. Notable risk factors for AAD include age, hypertension, smoking, aneurysm, and atherosclerosis [63,67,68,69,70,71,72,73] as well as congenital bicuspid aortic valve and genetic disorders, such as Marfan or Loeys–Dietz syndrome, which affect the connective tissue within the arterial wall [65,67]. Besides, 80% of patients with AAD suffer from hypertension [65] and show elevated levels of pro-inflammatory cytokines [74]. 

The striking point assuming the potential role of baroreceptors in AD and AAD is uncontrolled systemic hypertension, which is not uncommon in patients with AAD, as achieving effective blood pressure control is difficult [75,76]. Standard antihypertensive therapy is often inadequate, which could hypothetically be attributed to the attenuated baroreflex sensitivity in these patients. Consequently, AD can be associated with baroreceptor reflex failure resulting in hypertension and subsequent dysregulation of the blood pressure maintenance [60,77]. Dissection of the aorta might disrupt signal transduction of blood pressure changes, reflected by shear stress transferred across the aortic wall, towards the mechanosensory baroreceptors in the outer layer of the aortic arch. Without the physiological blood pressure and the pulsatile flow inside of the artery, extracellular matrix remodeling is initiated [78], leading to weakening of the aortic wall and vascular cell depletion. Besides, the undocking of the adventitial part in AD leads to unwanted ECM remodeling around the baroreceptors affecting their sensitivity.

Taken together, there are still many gaps in understanding of the molecular mechanism of the development and pathological progression of AD, including an apparent loss of SMCs and the dynamic imbalance of the individual extracellular matrix (ECM) components. This lack of knowledge limits the identification of potential therapeutic targets. Moreover, the effects of dissection on baroreceptor sensitivity and/or AD-associated changes in baroreceptor expression have not yet been investigated.

## 5. Baroreceptors and Aortic Aneurysms

Aortic aneurysm (AA) is a life-threatening clinical condition, characterized as a pathological expansion of the aorta, particularly in an elderly population (>65 years) [79,80,81,82,83]. Proteolytic degradation of the extracellular matrix (ECM) is thereby the main factor weakening the aortic wall, leading to diameter expansion and finally to rupture [81,82]. The increase in aortic diameter as well as the changes in the mechanical properties of the aortic wall may also significantly affect the function and sensitivity of baroreceptors. For instance, Klassen et al. [84] showed that vascular mechanics of the AA significantly contribute to differences in baroreflex modulation of the heart rate. However, as described by Lord et al. [85], wall tension seems to be more appropriate than aortic diameter alone, as both pressure and diameter have an influence on baroreflex function. More importantly, absolute diameter is not helpful; it particularly shows the change from diastolic-systolic diameter, tension to quantify loading and the subsequent systolic-diastolic change diameter, and tension to quantify unloading. These nuances of baroreflex regulation are yet to be characterized. Thus, the baroreceptor sensitivity should be determined via the vessel diameter and not by measuring the blood pressure. However, such measurements have not yet been performed. 

In the context of baroreceptors, no study has so far dealt with their role in an aneurysm in humans. Only Colney et al. [59] posed the hypothesis of potential existence of a multi-node baroreceptor network that measures blood pressure at all arterial bifurcations and thus allows a system-wide hemodynamic and vasomotor regulation. Failure in these putative baroreceptors might explain the non-optimal long-term outcomes by current surgical or endovascular treatments of aortic aneurysm. The work of Colney et al. [59] provides an interesting basis for the understanding of thoracic and abdominal aortic aneurysm. Presuming the existence of such putative baroreceptors at arterial bifurcations would lead to novel therapeutic strategies. However, it must be mentioned that such a hypothesis has not yet been proven.

## 6. Conclusions

Despite extensive research, the nature of baroreceptors and their mechanotransduction in humans remains elusive. In particular, all molecular and morphopathological investigations have been performed in animal studies. Neuronal control of blood pressure through baroreceptors in the aortic arch seems to be an essential feature of circulatory control. Arterial baroreflex is a key regulator of blood pressure, and its dysfunction has been linked with several cardiovascular diseases. Aortic aneurysms and dissection of the ascending aorta as well as aging could significantly affect baroreflex sensitivity, which might in turn lead to hypertension and anergy against blood pressure-lowering drugs. Furthermore, genetic disorders, such as Marfan or Loeys–Dietz syndrome, and congenital bicuspid aortic valve affect the connective tissue within the arterial wall and significantly increase the risk of AD. Thus, elucidating the precise molecular nature of baroreceptors and their signaling as well as characterizing the mechanisms underlying age- and disease-associated changes in baroreceptor sensitivity within the dissected aorta are of utmost clinical importance. Achieving effective blood pressure control in AD and AAD is challenging, leading to a high incidence of uncontrolled systemic hypertension; the ineffective therapies do suggest that baroreceptor dysfunction may contribute to the arterial hypertension associated with AD and AAD.

## Data Availability

Not applicable.
